# A Novel Network Profiling Analysis Reveals System Changes in Epithelial-Mesenchymal Transition

**DOI:** 10.1371/journal.pone.0020804

**Published:** 2011-06-07

**Authors:** Teppei Shimamura, Seiya Imoto, Yukako Shimada, Yasuyuki Hosono, Atsushi Niida, Masao Nagasaki, Rui Yamaguchi, Takashi Takahashi, Satoru Miyano

**Affiliations:** 1 Human Genome Center, Institute of Medical Science, University of Tokyo, Minato-ku, Tokyo, Japan; 2 Nagoya University Graduate School of Medicine, Showa-ku, Nagoya, Japan; National Cancer Institute, United States of America

## Abstract

Patient-specific analysis of molecular networks is a promising strategy for making individual risk predictions and treatment decisions in cancer therapy. Although systems biology allows the gene network of a cell to be reconstructed from clinical gene expression data, traditional methods, such as Bayesian networks, only provide an averaged network for all samples. Therefore, these methods cannot reveal patient-specific differences in molecular networks during cancer progression. In this study, we developed a novel statistical method called NetworkProfiler, which infers patient-specific gene regulatory networks for a specific clinical characteristic, such as cancer progression, from gene expression data of cancer patients. We applied NetworkProfiler to microarray gene expression data from 762 cancer cell lines and extracted the system changes that were related to the epithelial-mesenchymal transition (EMT). Out of 1732 possible regulators of E-cadherin, a cell adhesion molecule that modulates the EMT, NetworkProfiler, identified 25 candidate regulators, of which about half have been experimentally verified in the literature. In addition, we used NetworkProfiler to predict EMT-dependent master regulators that enhanced cell adhesion, migration, invasion, and metastasis. In order to further evaluate the performance of NetworkProfiler, we selected Krueppel-like factor 5 (KLF5) from a list of the remaining candidate regulators of E-cadherin and conducted *in vitro* validation experiments. As a result, we found that knockdown of KLF5 by siRNA significantly decreased E-cadherin expression and induced morphological changes characteristic of EMT. In addition, *in vitro* experiments of a novel candidate EMT-related microRNA, miR-100, confirmed the involvement of miR-100 in several EMT-related aspects, which was consistent with the predictions obtained by NetworkProfiler.

## Introduction

Currently, several large-scale omics projects, such as the National Cancer Institute's Cancer Genome Atlas (http://cancergenome.nih.gov/) and the Sanger Institute's Cancer Genome Project (http://www.sanger.ac.uk/genetics/CGP/), produce large amounts of data, including genomic, epigenomic, and transcriptomic information, about cancer patients or cell lines. Two challenges in omics are to construct and analyze patient-specific molecular networks to develop a comprehensive understanding of the molecular mechanisms of tumorigenesis and to identify molecules that are critical for tumor proliferation and progression [1]. If these challenges can be overcome, it may be possible to personalize cancer therapy, improve its efficacy, and reduce its toxicity and cost [2], [3].

Systems biology integrates various types of omics data and computational tools to represent and analyze complex biological systems. For example, gene network estimation that is based on Bayesian networks or mutual information networks can reconstruct biological systems from gene expression data [4]. However, most traditional gene network estimation methods construct a static network by using gene expression data from different cellular conditions. As a result, these methods only produce an averaged network for all patients and cannot reveal patient-specific molecular mechanisms of cancer. In addition, it is very difficult to infer a patient-specific gene network from only a few gene expression profiles of the patient without making any assumptions about the network.

In this study, we developed a novel statistical method called NetworkProfiler, which infers patient-specific gene regulatory networks from a dataset of cancer gene expression profiles. NetworkProfiler is based on a statistical graphical model with varying coefficients and a kernel-based data integration method with elastic net regularization for parameter estimation. A key feature of NetworkProfiler is that the strengths of the relationships between genes are allowed to vary depending on cancer characteristics, such as cancer progression, metastasis, disease-free survival, and drug sensitivity. NetworkProfiler groups samples according to the specific cancer characteristics so that neighboring samples have common gene regulatory systems. Then, by integrating the gene expression profiles of neighboring samples with a kernel method, NetworkProfiler produces a gene regulatory network for each sample. Finally, we analyzed 2 post-analysis to discover upstream regulatory genes and downstream target genes for specific cancer characteristics. NetworkProfiler is the first algorithm for constructing patient-specific gene regulatory networks from clinical cancer gene expression data to elucidate cancer heterogeneity.

We applied NetworkProfiler to gene expression microarray data from 762 cancer cell lines to determine system changes related to the epithelial-mesenchymal transition (EMT). The epithelial-mesenchymal transition (EMT) is a process that changes proliferating cells from an aplanetic state to a motile state [5], which allows cancer cells to leave the primary tumor and metastasize. The loss of E-cadherin, a cell adhesion molecule, is a biomarker of EMT [5]. NetworkProfiler identified 25 key regulators of E-cadherin, of which half have been previously described and the other half were novel candidates. NetworkProfiler also revealed regulatory changes in *miR-141*, *ZEB1*, and E-cadherin. Specifically, our results suggested that decreased expression of *miR-141* in mesenchymal cells disrupts the negative feedback loop between *miR-141* and *ZEB1*, which would allow *ZEB1* to decrease the expression of E-cadherin during the EMT. In addition, we predicted 45 EMT-dependent putative master regulators that control sets of genes involved in cell adhesion, migration, invasion and metastasis, namely, 17 of which are downstream targets of TGFB1, a master switch of the EMT. To further validate the performance of NetworkProfiler, we experimentally evaluated *in silico* predictions obtained by NetworkProfiler. We consequently found that knockdown of KLF5, a new candidate regulator of E-cadherin, decreased E-cadherin expression and induced morphological changes characteristic of EMT. In addition, the functional involvement of miR-100 was validated in some EMT-related aspects, which was consistent with the predictions obtained by Network Profiler.

## Results

### Overview of NetworkProfiler

**Figure 1:**
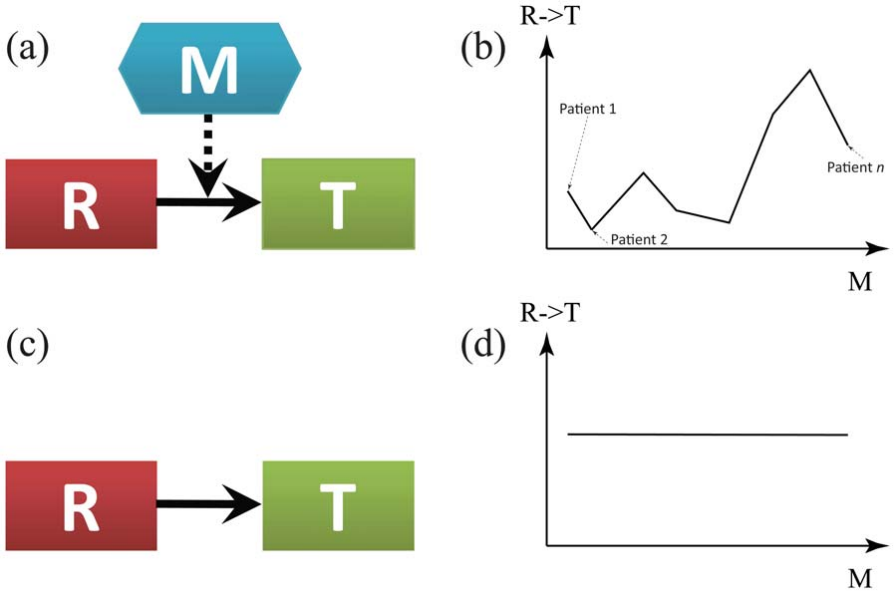
The relationships between a regulator (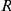), a target(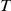), and a modulator (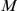) in NetworkProfiler and existing graphical models. (a). The relationships between 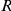, 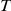 and 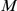 in NetworkProfiler. The directed solid-line edge from 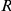 to 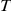 represents “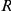 regulates the transcript of 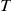
”. The directed dot-line edge from 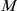 to the edge between 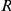 and 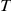 describes “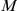 controls the strength of the relationship between 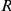 and 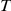
”. (b). The strength of the relationship between 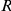 and 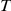 in NetworkProfiler that varies depending on the value of 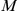. (c). The relationships between 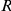 and 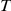 in existing graphical models that do not consider the effect of 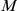. (d). The strength of the relationship between 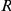 and 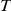 in existing graphical models that remains constant for all values of 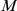.

Here, we provide an overview of NetworkProfiler; please refer to the Methods section for a complete description. NetworkProfiler is a modulator-dependent graphical model because it includes a modulator (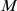) variable in addition to regulator (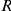) and target (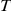) variables (genes). 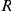 controls the transcription of 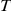 and 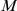 is a cofactor that modulates the interaction between 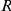 and 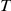. In this study, we defined 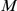 as a biological or a clinical feature that is related to cancer, such as drug response, survival risk, or a molecule or pathway that is related to cancer initiation, progression, or metastasis. The relationships between 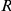, 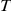, and 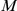 are illustrated in Figure 1a. As shown in Figure 1b, the strength of the relationship between 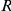 and 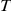 varies depending on the value of 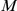. Thus, 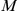 does not affect 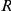 and 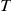 directly; instead, it influences the strength of the relationship between 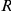 and 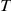. In contrast, existing graphical models, such as Bayesian networks and mutual information networks [4], do not consider the effect of 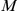 (Figure 1c), so the strength of the relationship between 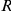 and 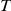 remains constant for all values of 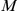 (Figure 1d).

**Figure 2:**
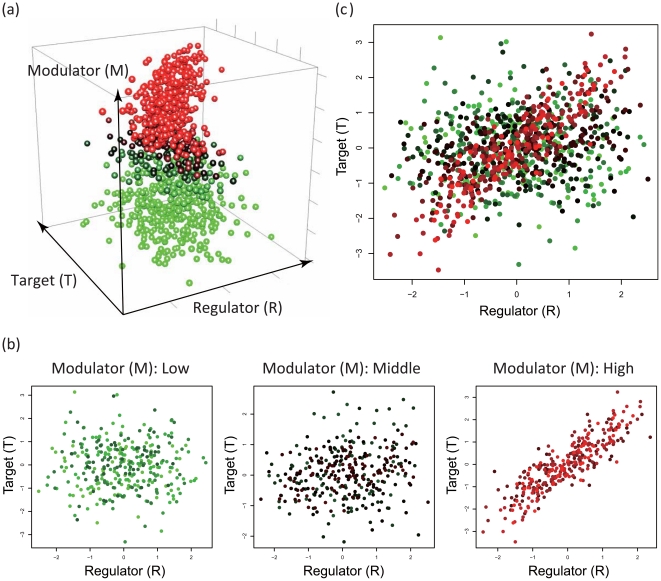
A regulatory change between a regulator (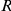) and a target (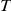) depending on the value of a modulator 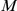. (a). A simple example with synthetic data from 1000 samples for 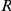, 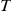, and 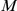 where 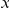-, 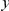-, and 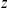-axises correspond to the expressions of 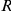 and 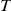, and the values of 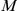, respectively. (b). The 3 scatter plots of 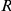 and 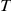 that are conditioned on the value of 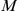. The left, middle, and right figures represent the scatter plots from 1-st sample to 333-th sample, from 334-th sample to 666-th sample, and from 667-th sample to 1000-th sample in order of ascending 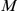, respectively. (c). The scatter plot of 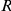 and 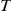 that are not conditioned on the value of 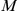.

In addition, NetworkProfiler can infer the relationships between 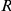 and 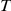, given a value of 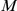. As a result, we could use NetworkProfiler to construct patient-specific networks with varying 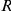-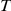 relationships that reflect changes in the feature of interest in cancer patients. A simple example with synthetic data for 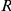, 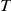, and 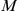 is shown in Figure 2a. In this example, we assume that 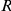 regulates 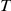 only with a high value of 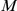 (Figure 2b). In this case, most existing methods that only consider 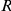 and 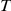 in all of the samples (Figure 2c) and ignore 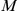 would conclude that 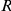 does not regulate 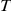. In contrast, NetworkProfiler attempts to quantify the strength of the relationship between 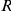 and 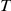 for a specific value 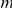 of 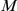 by reweighting the data according to the value of 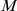 to identify the neighborhood of samples with values of 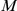 that are close to 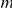. Then, NetworkProfiler measures the dependency between 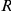 and 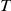 on the basis of these neighboring samples. The optimization of the size of the neighborhood is explained in the Method section.

**Figure 3:**
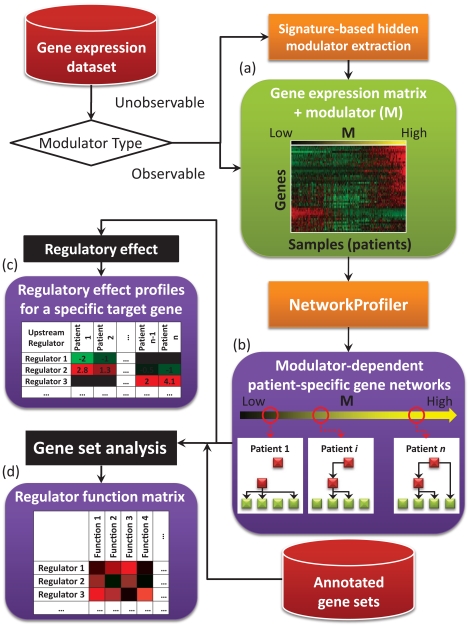
A schematic representation of the entire analytical process of NetworkProfiler. (a). Inputs of NetworkProfiler: gene expression data matrix and the modulator for each sample. (b). Outputs of NetworkProfiler: a set of gene networks for every value of 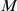 (i.e., sample-specific gene networks). (c). The regulatory effect profiles of the upstream regulators for a specific target gene. (d). The resulting regulator function matrix whose columns are the candidate regulators and rows are functions that are enhanced in the target genes.

A schematic representation of the entire analytical process of NetworkProfiler is shown in Figure 3. NetworkProfiler used 2 inputs: (1) gene expression data and (2) the modulator for each sample (Figure 3a). The gene expression data was represented as a 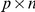 matrix, where 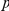 is the number of genes and 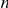 is the number of samples (patients). If the modulator was an observable variable, then we directly applied NetworkProfiler to these inputs. However, if the modulator was a variable that is difficult to observe, then we used a signature-based hidden modulator extraction algorithm to estimate the value of the modulator. The output of NetworkProfiler is a set of gene networks for every value of 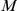 (i.e., sample-specific gene networks) shown in Figure 3b.

Afterwards, we used 2 post-analysis techniques to extract biological information from the networks. The first technique identified upstream regulators of a target gene of interest in the constructed modulator-dependent gene networks. To evaluate the modulator-dependent strength of a regulator for the target gene, we created a measure called the regulatory effect. The regulatory effect profiles of the upstream regulators for specific target genes are shown in Figure 3c. The second technique discovered putative master regulators that control downstream target gene sets with previously curated functions. To evaluate the enrichment of the target genes on a functional gene set, we created measure called the enrichment score. The resulting regulator-function matrix (Figure 3d) illustrates the candidate regulators (rows) of functions (columns) that are enhanced in the target genes.

### Identification of system changes in the epithelial-mesenchymal transition

To identify system changes during the EMT, we applied NetworkProfiler to gene expression profiles of 762 cancer cell lines from the Sanger Cell Line Project (http://www.broadinstitute.org/cgi-bin/cancer/datasets.cgi). This dataset included the expression profiles of 22,777 probes, which correspond to 13,006 mRNAs in these cancer cell lines from the Affymetrix GeneChip Human Genome U133 Array Set (HG-U133A) and the expression profiles of 502 human microRNAs from bead-based oligonucleotide arrays. The MAS5-normalized mRNA dataset was further transformed to the log scale and quantile-normalized. During the mapping of the probes to genes, we selected 1 probe for each gene that had the largest variance, which produced a final 13,508 (genes) 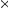 762 (cancer cell lines) gene expression matrix.

In this study, we considered transcription factors, nuclear receptors, and microRNAs to be potential regulators. To identify transcription factors and nuclear receptors, we selected human genes that were annotated as a “transcription regulator” or “ligand-dependent nuclear receptor” from the Ingenuity Knowledge Base (IKB; http://www.ingenuity.com). We also included some transcription factors that were not annotated in the IKB but were annotated in the Biobase Knowledge Library (BKL; http://biobase-international.com/). We mapped a total of 1230 genes in the HG-U133A microarray gene set to 1183 transcription factors and 47 nuclear receptors (Table S1). In addition, we included 502 human miRNA probes (Table S2).

To calculate the modulator values for the EMT in the 762 cancer cell lines, we applied a signature-based hidden modulator extraction algorithm (see Methods for details) to the expression data. First, we selected 122 genes labeled “EMT_UP”, “EMT_DN”, “JECHLINGER_EMT_UP”, and “JECHLINGER_EMT_DN” from Molecular Signatures Database v2.5 ([6]; http://www.broadinstitute.org/gsea/msigdb/index.jsp). Then, this algorithm narrowed the set to 50 functionally coherent genes with 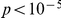 by using the extraction of expression module (EEM) [7] (Table S3) and computed the first principal component of these 50 genes as hidden values of the EMT-related modulator (Table S4). Since the direction of the first principal component did not always correspond to that of the EMT, we changed the sign of the modulator values by multiplying either plus or minus one so that epithelial-like cells have lower modulator values than mesenchymal-like cells.

**Figure 4:**
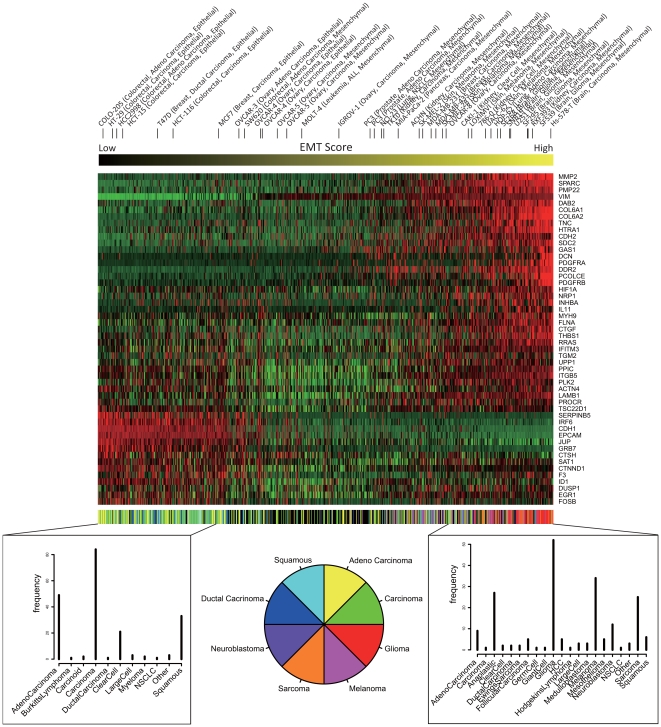
Expression profiles of the 50 functionally coherent genes in ascending order of the EMT-related modulator values. The heatmap represents normalized expression profiles so that the mean and variance for each gene are 0 and 1, respectively. The red color represents positive expressions and the green color represents negative expressions. The upper strings indicate cell line names which are known to be epithelial or mesenchymal. The upper horizontal color bar represents the values of the EMT-related modulator with the signature-based hidden modulator extraction algorithm. The bottom horizontal color bar shows primary histories of 762 cancer cell lines whose color corresponds to one of the eight primary histories or the other histories (black). The bottom histograms represent frequencies of the primary histories between samples with the 200 lowest and 200 highest values of the EMT-related modulator, respectively.

Figure 4 shows the expression profiles of the 50 functionally coherent genes in ascending order of the EMT-related modulator values. These modulator values clearly discriminated cell lines that were epithelial-like or mesenchymal-like. Specifically, cells with smaller or larger modulator values had more epithelial or mesenchymal phenotypes, respectively. Furthermore, many carcinomas and squamous tumors had low modulator values, while many gliomas and melanomas had high values. By using these EMT-related modulator values, NetworkProfiler constructed 762 regulatory gene networks that are related to the EMT. The list of the estimated edges in each of these networks can be downloaded from the supporting web site (Files S1, S2, and S3; http://bonsai.hgc.jp/ 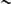
shima/NetworkProfiler).

### Identification of regulators of E-cadherin that induce the epithelial-mesenchymal transition

To identify possible regulators that might control the expression of E-cadherin during the EMT, we calculated the regulatory effects of the upstream regulators of E-cadherin. Out of 1732 potential regulators, NetworkProfiler inferred that 370 of them may control the expression of E-cadherin in any of the 762 cancer cell lines (Table S5). These putative regulators were ranked according to the change in their regulatory effect during the EMT. Although we did not include any information on known E-cadherin regulators, about half of the 25 highest ranked regulators were previously reported in the literature (Table 1). For example, 2 zinc finger transcription factors, ZEB1 and ZEB2, are direct repressors of E-cadherin and are involved in the EMT [9], . In addition, the miR-200 family indirectly suppresses the EMT by inhibiting the translation of ZEB1 and ZEB2 mRNAs [8]. Similarly, miR-192 inhibits the translation of ZEB2 [13], [14]. In addition, SNAI2, a member of the Snail superfamily of zinc finger transcription factors, also is involved in the EMT [16]. Likewise, TCF4 (also known as E2-2), a class I bHLH transcription factor, is an EMT regulator; its isoforms induce the EMT in MDCK kidney epithelial cells [12]. In contrast, FOXA1 and FOXA2 are positive regulators of E-cadherin, which suppress the EMT in pancreatic ductal adenocarcinoma [11]. KLF4 also inhibits the EMT by regulating E-cadherin expression [10]. NetworkProfiler also identified several other known direct repressors of E-cadherin, such as TWIST1 [17] and TCF3 (also known as E47) [18]; however, these regulators were ranked 38th and 84th, respectively.

**Table 1:** 25 top-ranked regulators of E-cadherin for the change in the regulatory effect change among the EMT with published evidence.

regulator	type	regulatory effect change	Evidence
IRF6	A	101.04	
miR-141	A	87.58	[8]
GRHL2	A	64.13	
ZEB1 (SIP1)	I	50.72	[9]
LSR	I	46.89	
miR-200b	A	31.55	[8]
KLF4	A	26.28	[10]
OVOL2	A	22.08	
miR-200a	A	17.70	[8]
FOXA2	A	17.26	[11]
TCF4 (E2.2)	I	14.15	[12]
ELF3	A	13.58	
ZNF217	A	13.53	
MYB	A	12.50	
KLF5	A	12.42	
miR-192	A	12.30	[13, 14]
FOXA1	A	11.69	[11]
ZNF165	A	11.39	
NKX2-1	A	11.21	
HNF1B	A	11.08	
TFE3	A	11.01	
ZEB2 ( 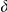 EF)	I	10.66	[15]
TRIM29	I	9.87	
SNAI2	I	9.74	[16]

The labels “A” and “I” indicate 2 types of the regulator: activator (A) and inhibitor (I). See Table S5 for the complete table of the 370 putative regulators for E-cadherin.

The other half of the 25 highest ranked regulators has not yet been reported and may be novel EMT-dependent regulators of E-cadherin. For example, although the relationship between GRHL2 and EMT is not known, GRHL2 is required for morphogenesis of epidermal and tracheal cells and plays an important role in regulating the expression levels of E-cadherin in *Drosophila* post-embryonic neuroblasts [19]. ZNF217 binds the E-cadherin promoter [20], which suggests that ZNF217 might be a transcription factor for E-cadherin.

**Figure 5:**
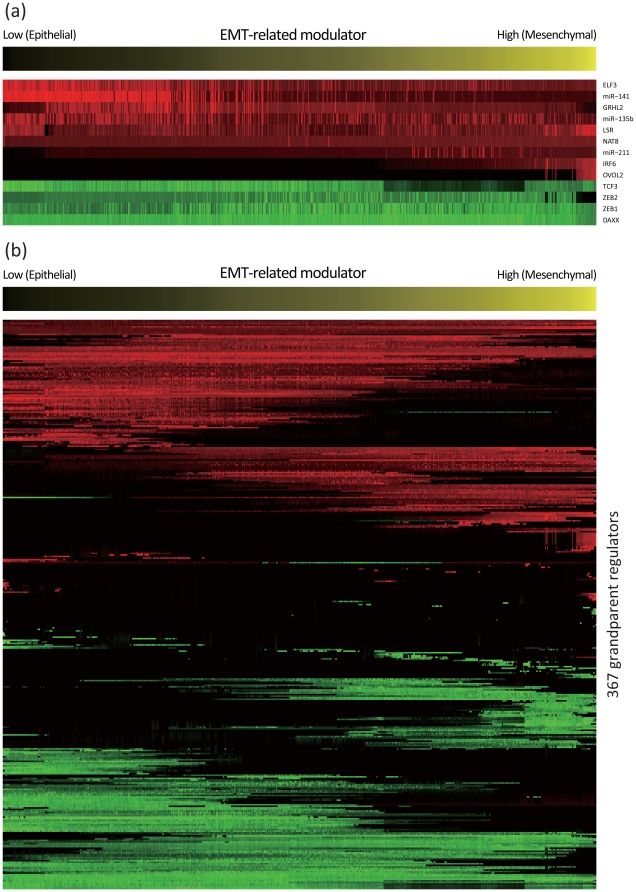
Regulatory effect profiles of the putative regulators of E-cadherin among the EMT. (a). The regulatory effect profiles of the 13 putative regulators among the EMT when the length of the paths from the regulators to E-cadherin is 1 where rows indicate the putative regulators of E-cadherin and columns indicate samples (cancer cell lines). The positive (red) and negative (green) regulatory effect indicate that the parent regulator controls the transcript of E-cadherin positively and negatively, respectively. (b). The regulatory effect profiles of the 13 putative regulators among the EMT when the length of the paths from the regulators to E-cadherin is 2.

Next, we compared the performance of NetworkProfiler with that of a structural equation model (SEM) of E-cadherin that was inferred by the elastic net [22]. This model was equivalent to a regression model where the response variable is the expression of E-cadherin and the explanatory variables are the 1732 regulator expressions. The significance of each regulator was evaluated based on the number of non-zero regression coefficients in 1000 bootstrapped datasets. The SEM inferred 627 putative regulators (Table S6). Among these putative regulators, there were only 6 regulators, namely, *ZEB1*, *miR-141*, *ZEB2*, *TCF3*, *miR-200b*, and *miR-200c*, in the 25 highest ranked regulators that were previously reported in the literature. This result suggested that NetworkProfiler was superior to the traditional gene network estimation methods to identify regulators of E-cadherin that are involved in the EMT. Moreover, NetworkProfiler can reveal regulatory changes among genes during the EMT. Figures 5a and 5b show the regulatory profiles of putative regulators of E-cadherin when the lengths of the paths from the regulators to E-cadherin is 1 and 2, respectively.

**Figure 6:**
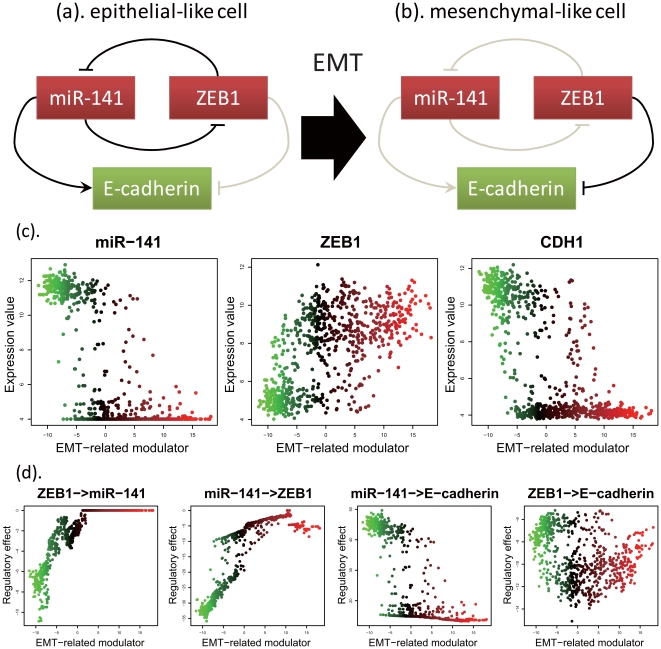
Regulatory changes among miR-141, ZEB1, and E-cadherin among the EMT. (a). The relationship among miR-141, ZEB1, and E-cadherin in epithelial-like cells. (b). The relationship among miR-141, ZEB1, and E-cadherin in mesenchymal-like cells. (c). The expression profiles of miR-141 (left), ZEB1 (middle), and E-cadherin (right) in order of ascending the EMT-related modulator values. The green and red colors indicate epithelial- and mesenchymal-like cells, respectively. (d). The regulatory effects from ZEB1 to miR-141, from miR-141 to ZEB1, from miR-141 to E-cadherin, and from ZEB1 to E-cadherin when the length of the paths is 1.

NetworkProfiler can also predict mechanistic interpretations of published experiments. For example, it is known that ZEB1 and ZEB2 induce EMT by repressing E-cadherin transcription and that ectopic expression of the miR-200 family (miR-200a, miR-200b, miR-200c, and miR-141) or miR-205 leads to downregulation of ZEB1 and ZEB2, upregulation of E-cadherin, and mesenchymal-epithelial transition (MET) in cells [8]. As the relationships between these genes, the prediction of NetworkProfiler provides the following results. As shown in Figures 6c and 6d, although the expression of miR-141 had a strong positive effect on that of E-cadherin in epithelial-like cells, this effect decreases during the EMT. In contrast, although the expression of ZEB1 had a weak negative effect on that of E-cadherin in epithelial-like cells, this effect increased during the EMT. Interestingly, miR-141 and ZEB1 had a strong, direct negative effect on each other only when the EMT-related modulator values were low. This implied that there is a negative feedback loop between miR-141 and ZEB1 in epithelial-like cells, which is consistent with a previous study [23]. Furthermore, during the EMT, the expression levels of miR-141 and E-cadherin decreased, while the expression level of ZEB1 increased. These results suggested that reduced expression of miR-141 disrupts the negative feedback loop between miR-141 and ZEB1 (Figures 6a and 6b), which would allow ZEB1 to decrease the expression of E-cadherin, as illustrated in Figure 6c. It should be noted that these results cannot be predicted by traditional graphical models which infer a static gene network structure.

### Identification of relationships between regulators and epithelial-mesenchymal transition-related functional gene sets

The EMT-dependent relationships between downstream target genes for each regulator and previously curated functional gene sets in each sample were analyzed by applying gene set analysis (see Methods for details) to the constructed gene networks for 762 cancer cell lines. We tested five curated gene sets included in Ingenuity Knowledge Base (IKB; http://www.ingenuity.com). These gene sets were related with *adhesion*, *migration*, *invasion*, and *metastasis* which were hallmarks of EMT [5], and EMT itself. By using gene set analysis, the statistical significances (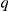-values) for the enrichments of downstream genes for the 1732 regulators on the five functional gene sets were calculated in each of the 762 cell lines. These results can be downloaded from the supporting web site (File S4; http://bonsai.hgc.jp/ 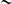
shima/NetworkProfiler).

To search for regulators that strongly affected the five EMT-related functional gene sets, the change in the enrichment score during the EMT and their integral 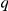-value were calculated. The result was summarized by a regulator function matrix (Table S7). We focused on 45 regulators with the integral 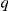-values less than 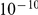 as putative master regulators that strongly enhanced the functional gene sets related with the EMT. Interestingly, among the 45 regulators, 17 regulators were downstream targets of transforming growth factor 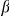-1 (TGFB1), a master switch of EMT [24], with published evidence (Table S8). This result suggests that these regulators have crucial roles in TGFB1-induced EMT.

As a control, we tested how well the NetworkProfiler analysis identified known relationships between regulators and functional gene sets in the Ingenuity Knowledge Base. The known functional relationships of the 45 putative master regulators are shown in Table 2. For example, FOSL1 increases the migration of MDA-MB-436 cells [25] and the invasion of A549 cells [26]. SMAD3 increases the adhesion [34], the metastasis [35], and the migration [36] of cells, respectively. Similarly, HIF1A increases the adhesion of undifferentiated trophoblast stem cells [27], the metastasis of LM2 cells [41], the migration of HUVEC cells [42], and the invasion of Achn cells [43], respectively.

**Table 2:** Selected relationships between the 47 putative master regulators and the 5 functional categories with published evidence.

regulator	function	- 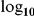 ( 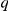 -value)	mode of action (E 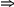 M)					evidence
			A 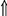	A 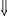	I 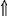	I 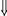	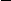	
FOSL1	migration	9.82	29	2	42	3	41	[25]
	invasion	8.42	14	2	24	3	22	[26]
EPAS1	adhesion	5.90	26	1	10	0	16	[27]
	migration	7.66	32	1	14	0	24	[28]
KLF5	migration	5.93	28	2	27	5	25	[29]
AHR	metastasis	3.67	12	0	11	0	9	[30]
FOXF1	metastasis	6.10	24	0	9	0	8	[31]
	migration	6.09	29	0	17	0	14	[32]
ELK3	migration	6.23	41	8	17	7	19	[33]
SMAD3	adhesion	4.57	9	3	23	0	10	[34]
	metastasis	3.12	5	1	12	1	9	[35]
	migration	5.24	14	5	26	1	21	[36]
	EMT	2.47	1	1	2	0	0	[37]
WWTR1	migration	5.08	32	0	17	3	16	[38]
	invasion	3.48	17	0	8	2	5	[38]
hsa-miR-145	invasion	2.52	13	0	8	3	17	[39]
CEBPD	metastasis	4.88	17	2	10	0	9	[31]
TGFB1I1	adhesion	5.12	25	2	23	5	11	[40]
HIF1A	adhesion	3.84	10	0	25	3	10	[27]
	metastasis	4.45	14	1	14	0	8	[41]
	migration	5.00	18	3	25	4	21	[42]
	invasion	3.65	12	0	9	3	10	[43]
SNAI2	migration	3.45	36	2	25	14	25	[25]
ELF3	adhesion	7.87	24	4	24	11	14	[44]
	invasion	4.45	9	3	18	6	21	[44]
SOX9	adhesion	6.80	18	2	19	0	26	[45]
	migration	5.46	28	2	15	1	23	[46]
GLI3	migration	4.53	24	7	24	7	26	[47]
TCF7L2	migration	4.52	19	10	18	1	27	[48]
NFKBIA	adhesion	2.73	12	2	14	3	12	[49]
	metastasis	2.39	5	0	5	3	9	[50]
	migration	3.98	18	2	18	7	23	[51]
	invasion	2.69	9	2	5	2	12	[50]
VAV1	adhesion	5.51	3	5	15	3	14	[52]
	migration	5.10	7	10	16	5	16	[53]
JUN	adhesion	3.03	15	4	6	5	6	[54]
	migration	3.31	19	2	7	7	14	[25]
	invasion	2.07	8	2	7	2	5	[55]
ETV1	invasion	2.50	13	1	13	5	7	[56]
PDLIM1	adhesion	4.27	16	6	17	6	29	[57]
MAFB	metastasis	4.41	9	0	3	8	6	[31]
GATA6	metastasis	3.25	11	3	4	1	4	[31]
RUNX1	adhesion	6.27	15	5	16	12	14	[58]
	migration	2.46	23	7	20	7	20	[59]
YAP1	migration	3.30	7	2	20	0	9	[60]

The labels “A
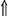
”, “A
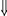
”, “I
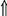
”, and “I
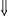
”, and “
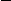
” indicate the number of the five modulator modes of action for the relationship between a regulator and its target included in the functional gene set: “the activation of a regulator on the expressions of its target genes with the functional category was increased by the modulator”, “inhibition increased”, “activation decreased”, “inhibition decreased”, and “the modulator mode of action is not determined”, respectively.

Although some of the 47 putative master regulators have not been reported to enhance the EMT-related functions in IKB, some predictions were supported by other resent works which were not included in IKB. For example, the prediction of NetworkProfiler suggested that PTRF regulates gene sets related with migration (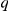-value = 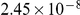) and with metastasis (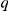-value = 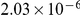) during the EMT. Consistent with the *in silico* result, PTRF expression inhibits migration and correlates with metastasis in PC3 prostate cancer cells [61]. Similarly, NetworkProfiler predicted that miR-146 contributes to migration (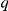-value = 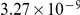) and invasion (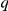-value = 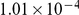) during the EMT. This *in silico* result is comparable with the *in vitro* result that miR-146 inhibits invasion and migration, and acts as a metastasis suppressor [62]. In addition, some predictions between miRNAs and functions seem reasonable based on the known functions of the miRNA host genes. For example, the prediction of NetworkProfiler provided the hypothesis that miR-143 and miR-145 promotes metastasis (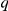-value = 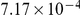 and 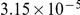) and migration (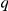-value = 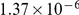 and 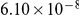), respectively. miR-143 and miR-145 cooperatively target a network of transcription factors, such as KLF4, to control smooth muscle phenotype switching [63]. Since KLF4 increases the migration of cells [29] and induces EMT [10], these miRNAs might be related with EMT-related functions or control EMT by targeting KLF4. Again, it should be noted that these relationships between regulators and functions cannot be predicted from one gene network constructed by traditional graphical models, and only the results of multiple network comparison between epithelial-like and mesenchymal-like cells based on NetworkProfiler enables us to support the recent biological knowledge and new hypotheses about unknown relationships.

### Comparison between *in silico* predictions and *in vitro* results

**Figure 7:**
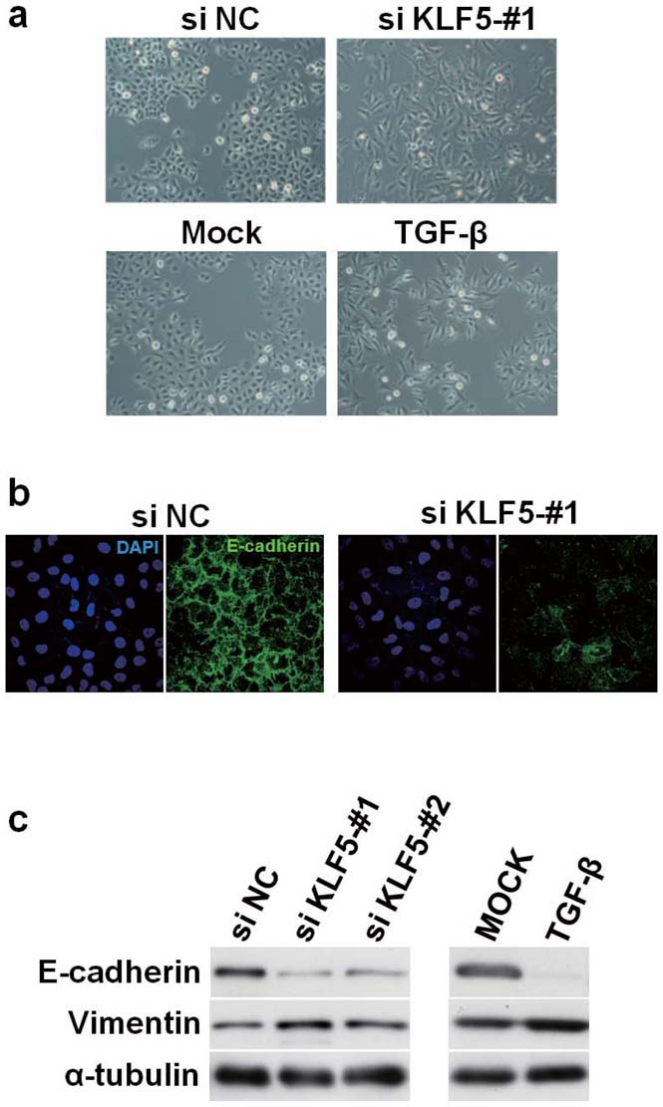
Induction of EMT by KLF5 knockdown in A549 NSCLC cell line. (a) Phase contrast images of A549 cells 72 hours after siRNA transfection, showing a fibroblast-like morphology in siKLF5 treated cells. TGF-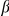 treatment serves as a positive control for EMT induction in A549 cells. (b) Representative immunofluorescence staining images, showing reduced E-cadherin expression in siKLF5-treated A549 cells. (c) Western blot analysis of E-cadherin and vimentin, showing EMT-related changes in their expression in A549 cells treated with two differenct siRNAs.

To validate the performance of NetworkProfiler, *in silico* predictions obtained by NetworkProfiler were evaluated experimentally. We first conducted *in vitro* experiments of a new candidate regulator of E-cadherin listed in Table 1, KLF5, to investigate whether KLF5 affects E-cadherin expression and induces morphologic changes characteristic of EMT using A549 lung adenocarcinoma cell line, which is well known to exhibit EMT in response to TGF-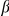 [64]. KLF5 knockdown markedly altered a cobblestone epithelial morphology of A549 cells and induced a more fibroblast-like morphology with reduced cell-cell contact, which was similar to that seen in TGF-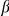-treated A549 cells (Figure 7a and Figure S1). Immunofluorescence analysis showed significant reduction of E-cadherin expression in A549 cells knocked down for KLF5 (Figure 7b), which was also confirmed by western blot analysis (Figure 7c). Conversely, vimentin expression was shown to be modestly increased by siKLF5 treatment (Figure 7c). Consistent with the *in vitro* results, the prediction of NetworkProfiler suggested that KLF5 affects E-cadherin expression as well as Vimentin expression during the EMT, since the changes in the regulatory effects from KLF5 to E-cadherin and Vimentin were much larger compared with the other regulators (12.42 and 16.57, respectively) which was ranked 15-th and 10-th among the 1732 regulators (Table S9). The result of gene set analysis (Table S7) also suggested that KLF5 affects EMT (q-value = 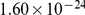). Thus, we consequently found that *in silico* predictions obtained by NetworkProfiler was confirmed with the results of *in vitro* experiments; KLF5, a newly identified candidate regulator of EMT, was shown to affect expressions of E-cadherin and Vimentin as well as morphologic characteristics related to EMT as a repressor of EMT.

**Figure 8:**
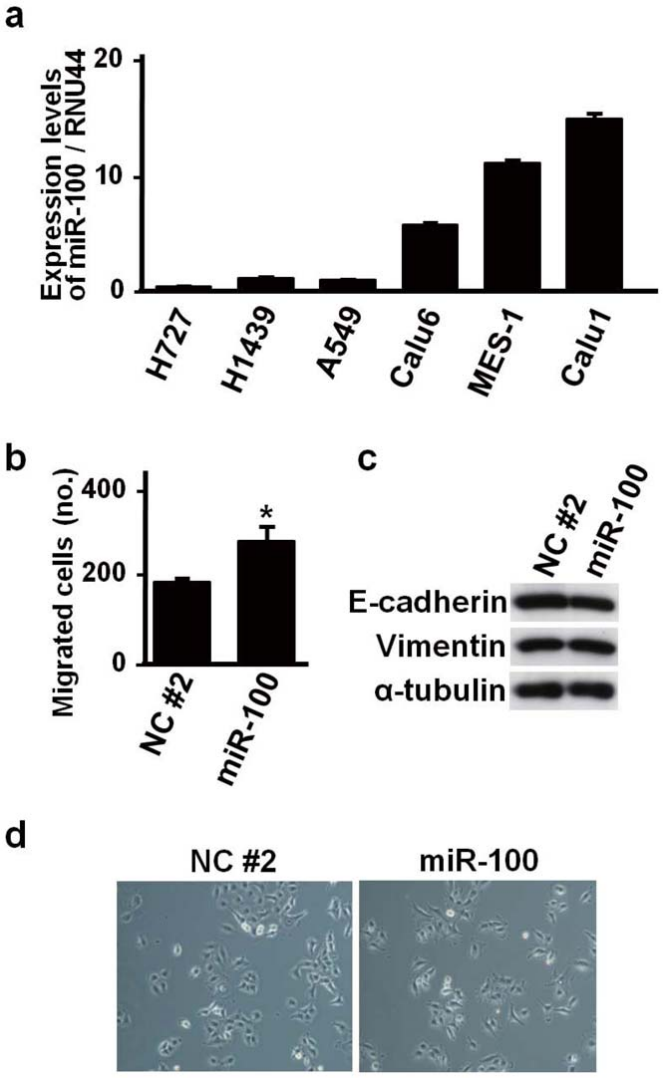
miR-100-induced changes in biologic characteristics in A549 NSCLC cell line. (a) Quantitative real-time RT-PCR analysis of miR-100 in six NSCLC cell lines, showing low miR-100 expression in A549, NCI-H727 and NCI-H1437. (b) Motility assay showing increased migration in miR-100-transfected A549 cells. Error bars indicate SE in three independent experiments (*, 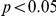). NC#2, negative control. (c) Western blot analysis of E-cadherin, vimentin and 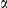-tubulin, showing lack of noticeable changes in miR-100-transfected A549 cells (d) Representative phase contrast microscopic images showing negligible changes in miR-100-trasfected A549 cells.

We also conducted *in vitro* experiments to validate functional involvement of a novel candidate EMT-related microRNA, miR-100 whose expression was increased in 762 cancer cell lines during the EMT (Figure S2). miR-100 was found to be expressed at a low level in A549, NCI-H727 and NCI-H1439 NSCLC cell lines, which had low EMT-related modulator values among the 762 cell lines panel (Figure 8a). miR-100 was transiently introduced into A549 cells, resulting in a significant increase of cell migration activity (Figure 8b). However, overexpression of miR-100 did not affect expressions of an epithelial marker, E-cadherin, and a mesenchymal marker, vimentin (Figure 8c), and also did not influence cell morphology (Figure 8d). However, overexpression of miR-100 significantly increased cell migration without noticeably affecting morphology in NCI-H727 and NCI-H1437 cells (Figure S3). Consistent with the *in vitro* results, the prediction of NetworkProfiler suggested that miR-100 enhances migration (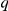-value = 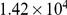) but does not affect EMT itself (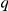-value = 0.24) from gene set analysis (Table S7). It also suggested that miR-100 does not affect the expressions of E-cadherin and Vimentin during the EMT, since E-cadherin and Vimentin were not target genes of miR-100 in all the 762 cell line-specific gene networks related with the EMT(Files S1, S2, and S3) and the changes in the regulatory effects from miR-100 to E-cadherin and Vimentin were much smaller compared with the other regulators (0 and 1.72, respectively), which were ranked 371-th and 151-th among the 1732 regulators (Table S9). Thus, we conclude that several hypotheses of miR-100 functions provided by NetworkProfiler are consistent with the results of *in vitro* experiments; NetworkProfiler has the potential to uncover novel biological mechanisms.

## Discussion

We developed a novel algorithm called NetworkProfiler to infer patient-specific, modulator-dependent gene regulatory networks from gene expression data. Unlike traditional methods that infer a static network for a specific state of a cell or an averaged network for many patients, NetworkProfiler can be used to construct patient-specific gene networks for specific diseases, such as cancer. Subsequently, information about the regulatory effects of individual genes and functional gene sets can be extracted from these networks. In order to show the performance of NetworkProfiler, we applied NetworkProfiler to microarray gene expression data from 762 cancer cell lines to identify the system changes that were related to the EMT. As a result, we identified 25 EMT-dependent regulators of E-cadherin. Although some of these regulators have been reported in the literature, others may be novel master regulators of E-cadherin that induce the EMT. Moreover, in comparison to the traditional SEM approach, the performance of NetworkProfiler was superior for identifying regulators of E-cadherin during the EMT. We also showed that NetworkProfiler can reveal regulatory changes of E-cadherin during the EMT. In particular, our results suggested that decreased expression of miR-141 disrupts the negative feedback loop between miR-141 and ZEB1, which would allow ZEB1 to decrease the expression of E-cadherin.

Furthermore, we also identified putative relationships between regulators and EMT-dependent functional gene sets, some of which had published evidence. Based on the significance of the enrichment of downstream target genes for the regulator on the 5 functional gene sets, we identified 45 putative master regulators for the EMT. We found that 17 regulators were downstream targets of TGFB1 that is a master switch of the EMT. We then showed that NetworkProfiler can not only predict the relationships between these regulators and functions that were supported by many published evidence, but also produce new hypotheses that some of them might enhance EMT-related functions or induce EMT.

Finally, it is of note that we were able to validate the *in silico* predictions obtained by NetworkProfiler in our *in vitro* experiments. KLF5, a newly identified candidate regulator of EMT, was experimentally shown to affect E-cadherin expression as well as morphologic characteristics related to EMT, validating the NetworkProfiler-based prediction that KLF5 is a negative regulator of EMT. We also conducted *in vitro* experiments of another regulator, miR-100, for which NetworkProfiler predicted its association with some EMT-associated functions. As a result, we found that the predicted miR-100 functions conformed to the results of *in vitro* experiments. Thus, we conclude that the effectiveness of the proposed method was validated not only from published literature but also from new *in vitro* validation experiments.

We anticipate several possible applications and extensions of NetworkProfiler. In this study, we only focused on the system changes that are associated with the EMT. NetworkProfiler also could be used to infer system changes and reconstruct modulator-dependent gene networks for other well-defined modulators, such as drug sensitivity and prognosis risk. Currently, a significant limitation of NetworkProfiler is that the modulator must be one-dimensional. However, cancer development is a multivariate process. It may be possible to use multivariate kernel functions in NetworkProfiler to overcome this limitation.

During the past decade, cancer therapy has become increasingly personalized [2], [3]. Unlike the traditional “one-size-fits-all” approach to cancer therapy, patient-specific cancer therapy reduces the side effects of chemotherapy and predicts the odds of cancer recurrence more accurately by tailoring cancer treatment to specific genetic defects in the tumors of individual patients. However, this goal is not an easy task since cancer is an extremely complex and heterogeneous disease. We believe that NetworkProfiler will help elucidate the systems biology of cancer and facilitate personalized chemotherapy.

## Materials and Methods

### Cell lines and reagents

Human non-small cell lung cancer (NSCLC) cell lines, A549, NCI-H1437 and NCI-H727, were purchased from American Tissue Culture Collection, while other NSCLC cell lines, Calu1, Calu6 and SK-MES1, were generously provided by Dr. L. J. Old (Memorial Sloan-Kettering Cancer Center). Cells were maintained in RPMI 1640 supplemented with 10% fetal bovine serum. The anti-E-cadherin antibody was purchased from BD Transduction Laboratories, anti-vimentin from Santa Cruz Biotechnology, anti-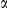-tublin from Sigma Aldrich, and anti-mouse IgG from Cell Signaling Technology. The Alexa-conjugated anti-mouse IgG was purchased from Molecular Probes. siRNAs against KLF5 (siKLF5 #1 and #2) and a negative control (siNC) were purchased from Sigma Genosys. Pre-miR has-miR-100 and negative control #2 were purchased from Ambion. Human TGF-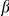 was purchased from R&D Systems, Inc.

### Immunostaining, western blot analysis and in vitro motility assay


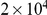
 cells in 6-well plates were transiently transfected with either 20 nM siRNA or 10 nM Pre-miR molecules using Lipofectamine RNAiMAX (Invitrogen), as previously described [65]. Immunofluorescence staining was carried out after fixation with 3.7% formaldehyde and postfixing with 0.1% Triton X-100 each for 10 min at RT. Photographs were taken 72 hr after transfection. Cells were harvested 48 hr after transfection for western blot analysis. In vitro motility assay based on Transwell-chamber culture systems was performed, as previously described [66].

### Quantitative real-time reverse transcription (RT)-PCR analysis

Quantitative real-time RT-PCR analysis of KLF5 was performed using Power SYBR Green (Applied Biosystems) and the following PCR primers:

5′-CCCTTGCACATACACAATGC-3′ and 5′-GGATGGAGGTGGGGTTAAAT-3′. Quantitative real-time RT-PCR analysis of miR-100 and RNU44 was performed using TaqMan probes and 7500 Fast Real-Time PCR system (Applied Biosystems), essentially as previously described [67].

### NetworkProfiler

NetworkProfiler employed a varying-coefficient structural equation model (SEM) to represent the modulator-dependent conditional independence between gene transcripts. Let there be q possible regulators, 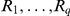, that may control the transcription of the 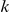-th target gene 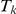 when the modulator 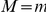. Then the varying-coefficient structural equation model for 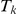 is
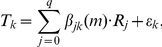
where 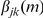 is the coefficient function that represents the effect of 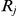 on 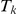, 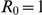, and 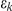 is a noise term. If 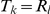, then the term 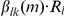 can be omitted from the model, i.e., 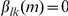 for all 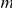. By estimating the parameters 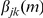, we obtain the transcriptional regulatory gene network at 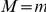.

We used a kernel-based method to estimate these parameters. Let there be 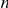 sets of gene expression profiles. Then, the SEM for the 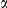-th sample can be rewritten as
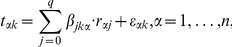
where 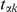, 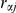, and 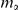 are the values of the 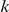-th target gene, the 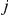-th regulator, and the modulator for the 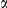-th sample, respectively; 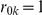, and 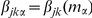. For 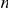 samples, we obtain 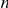 modulator-dependent gene regulatory networks, i.e., the regulatory effects of 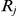 (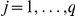) on 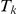 (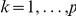) are determined by 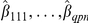, where 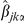 is the estimate of 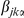.

We assumed that the values of the coefficients are almost constant for the neighborhood samples of the 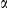-th sample with respect to the modulator 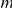, that is, 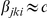 for the 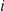-th sample that satisfies 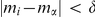 for some constant 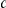 and small 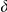. Then, we estimated the parameters 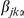 for fixed 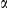 by minimizing a regularized kernel-based weighted residual sum of squares
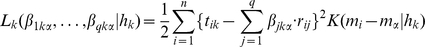


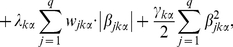
(1)where 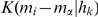 is a Gaussian kernel function defined by
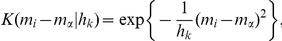
and 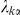 and 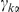 are hyperparameters that control the 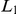 (lasso [68]) and 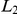 (ridge [69]) penalties, respectively. In addition, 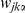 is an importance weight for 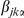, and 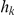 is the bandwidth of the Gaussian kernel. The kernel function 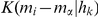 defines the neighborhood around the 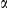-th sample in terms of 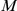; a large value of 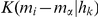 means that the 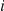-th sample is in the neighborhood of the 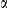-th sample. By fixing 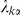, 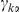, 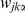, and 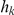, we obtain the estimates
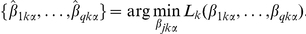



This parameter estimation method is a weighted version of the elastic net [22]. The 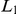 penalty zeroes some coefficients [68], which produces a sparse network structure. In contrast, the 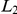 penalty stabilizes the solution by a grouping effect that promotes the collective inclusion or exclusion of highly correlated variables in the model [22]. The importance weights 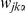 allow tuning parameters to take on different values for different coefficients 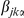. For example, if 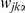 has a large value, then an estimator 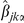 tends to be zero. In contrast, if 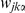 has a small value that is nearly equal to zero, 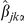 tends to be non-zero. These weights create a sparser network structure than the lasso and elastic net methods. The parameters 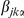 were estimated by using a recursive procedure, and the weights 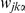 were updated by 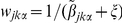 [70], where 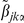 is the estimate from the previous step and 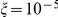 to avoid dividing by zero. Then, the modulator-dependent networks for 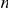 samples can be derived from the estimates of 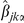 (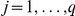, 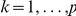, and 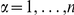).

For convenience of subsequent explanations, we introduce the following notations:
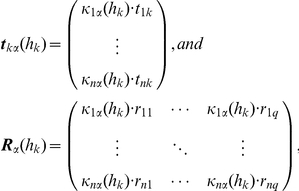
where 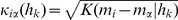.

In these expressions, 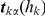 and 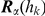 were normalized so that the means and variances for 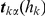 and each column of 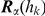 were 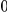 and 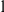, respectively. As a result, the intercept 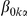 was not included in the loss function (1). For fixed 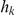, the loss function (1) can be minimized by using a kernel-based weighted version of the recursive elastic net [70]. The tuning parameters 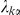 and 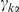 were selected by minimizing a modified version of the bias-corrected weighted Akaike information criterion (AIC) [71]:



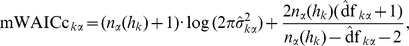
where 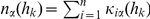, and 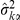 is estimated by
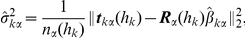
with 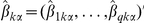. In addition, 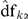 is the unbiased estimate of the degrees of freedom given by
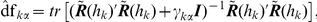
where 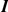 is the identify matrix and 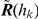 is the submatrix of 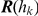, which has columns that correspond to the nonzero coefficients, respectively.

The NetworkProfiler algorithm is shown below:

#### Algorithm: NetworkProfiler

1: 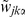 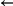 1 (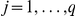)

2: iter 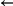 1

3: **for** 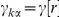 (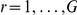) **do**

4: **repeat**

5: Calculate 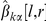 and 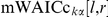 corresponding to 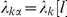 (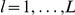).

6: 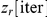 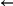 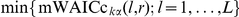


7: 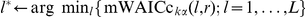


8: **if** 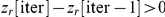 **then**

9: Exit loop

10: **else**

11: 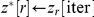


12: 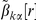 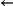 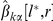


13: 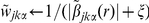 (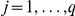)

14: 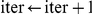


15: **end if**

**16: untill** iter reaches to 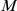.

**17: end for**

18: 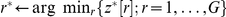


19: Return the coefficient vector 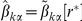.

The results from NetworkProfiler, which are the estimates of 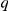 coefficients 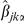 (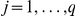) for the 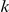-th target gene of the 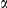-th patient, depend on the values of 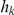. We used cross-validation to select an optimal value of 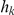 and estimate 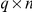 coefficients, 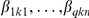 by minimizing the cross-validation error:
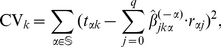
(2)


where 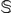 is a randomly selected set of samples and 
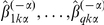
 are estimated from the remaining samples by minimizing:
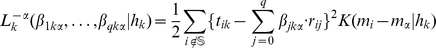


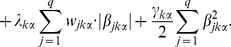
(3)


The algorithm in NetworkProfiler for minimizing this loss function (3) is shown below:

#### Algorithm: Conditional optimization with cross-validation

1: **for** 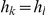 (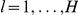) **do**

2: **for all** 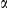 such that 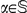 **do**

3: Calculate 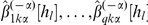 with NetworkProfiler.

4: **end for**

5: Calculate 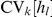.

6: **end for**

7: 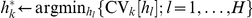


8: **for** 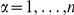 **do**

9: Calculate 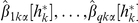 with NetworkProfiler.

10: **end for**

11: Return a sequence of the coefficient vectors 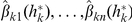.

Subsequently, the modulator-dependent gene networks for 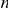 samples are determined from the coefficient vectors 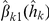, 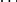, 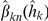 (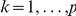) by applying the above algorithm for all 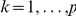. The computational cost of this algorithm rapidly increases as the number of samples and genes increase. Thus, for computers that only have a single central processing unit (CPU), this algorithm is only practical for medium-sized networks with up to several genes. However, since this algorithm can be executed in parallel for every 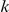, it can be run on a stand-alone workstation with multi-core CPUs and computer clusters. Figure S4 represents the histogram of computational times based on 12 core CPUs (Intel Xeon Processor E5450 (# of cores = 4, clock speed = 3.0GHz) 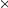 3) for calculating 762 cancer cell line-specific gene networks from 13,508 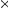 762 gene expression data through 100,000 iterations when 100 target genes were randomly selected among 13,508 genes and the number of regulators was not restricted, i.e., 1732 regulators were used. The average computational time was about 9 days. In this situation, we can find putative master regulators of the focused target genes related with a modulator of interest. Of course, for calculating gene networks of 762 samples for a large number of target genes, a supercomputer is required. In this study, we used the Super Computer System at the Human Genome Center, Institute of Medical Science, University of Tokyo, Japan, to analyze 762 gene networks with 13,508 target genes.

### Signature-based hidden modulator extraction

When the modulator was a variable that is difficult to observe, we used a signature-based hidden modulator extraction algorithm to estimate the value of the modulator for each sample. This algorithm takes seed genes that are related to the modulator and computes the underlying latent variable of the modulator by using principal components and extraction of expression modules (EEM) [7]. Let 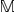 be a gene set that is related to the modulator and let 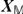 be an 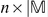 matrix of 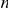 expression levels of 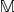. Then, a linear model, which is a special case of the single factor model [72], relates 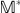, a subset of 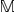, to an underlying latent variable 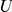 as follows:



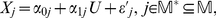
(4)where 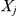 is the expression level of the 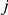-th gene in 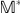, 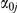 is the y-intercept, 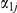 is a coefficient, and 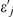 is a noise term. We assumed that other genes that do not include 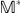 (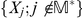) are independent of 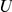.

The values of 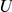 for 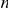 samples, 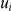 (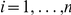), can be estimated by the following procedure:

#### Algorithm: signature-based hidden modulator extraction

1: For a given set 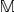, find a subset 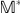 based on the expression coherence with the EEM algorithm [7].

2: Given 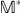, singular value decomposition of the data matrix 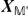 estimates 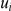 by the largest principal component.

3: Return the values 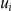 (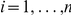).

In the first step, we estimate 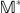. In the second step, we assume that the noise terms 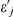 have Gaussian distributions with equal variances. As a result, the singular value decomposition generates maximum likelihood estimates of 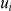 for the single factor model [72].

### Regulatory effect

To identify upstream regulators that had strong effects on the expression of a target gene of interest in the constructed modulator-dependent gene networks, we defined a measure, called the regulatory effect, of the effect of the 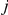-th regulator on the 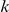-th target gene in the 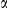-th sample as
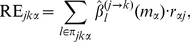
(5)where 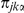 is the set of all possible paths from 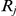 to 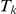, and 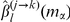 is the product of the estimated coefficients on the 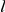-th path that includes 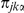. For example, given all the possible paths from 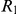 to 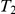 in the 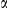-th sample (Figure S5), the set 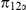 is
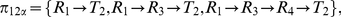
(6)and the regulatory effect 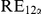 is
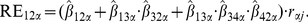
(7)


In our analysis, the length of the paths from 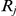 to 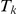 is restricted to either 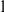 or 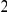.

To determine how the modulator affects the regulatory effect 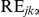, we also defined the change in the regulatory effect of the 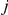-th regulator on the 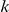-th target as
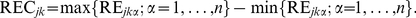
(8)


In addition to this definition, it is also possible to use percentiles instead of max and min to achieve more robust results. However, in our analysis, we used max and min to increase the power of the method. It should be noted that the change in the regulatory effect 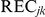 does not explain the mode of action for the modulator with respect to the regulator-target relationship. File S5 (http://bonsai.hgc.jp/~shima/NetworkProfiler) is provided to determine the modulator mode of action by statistical test.

### Gene set analysis of downstream genes for a regulator

To identify regulators that enhanced the functions of their targets, we calculated the statistical significance of the enrichment of targets for a given regulator in each sample. To test the enrichment, we use the degree of independence between the two properties:


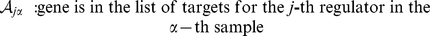
 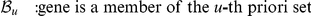


Testing the association between the properties 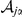 and 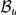 corresponds to Fisher's exact test. The 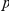-value calculated by this test, 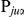, indicates the probability of observing at least the same amount of enrichment when downstream genes are randomly selected out of all genes. Thus, a very small 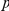-value gives strong evidence for an association between 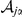 and 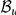 for the 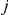-th regulator in the 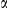-th sample. To correct for multiple hypotheses testing, Benjamini-Hochberg (BH)-corrected 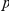-values (q-values) [73], 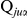, were calculated.

To determine how the modulator affects the functions of downstream genes for a regulator, we defined the enrichment score, 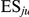, as a change in the statistical significance of the enrichment of targets for the 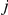-th regulator on the 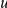-th function:
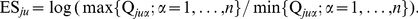
(9)


Thus, a very large 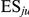 indicates that the modulator causes a significant change of the enrichment of the targets for the 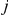-th regulator on the 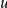-th function.

To identify putative master regulators that control more functional gene sets than other regulators, we also calculated the total enrichment score, 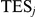, by combining independent enrichment scores, 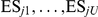, where 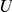 is the number of functional gene sets:
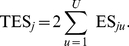
(10)


The total enrichment score is equivalent to the difference of the Fisher's statistic 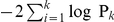 [74] which was used to combine independent tests obtained from 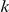 studies based on the 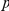-values, 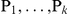. The Fisher's method is based on the fact that the statistic 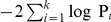 follows a chi-square distribution with 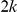 degrees of freedom under the global null hypothesis that all null hypotheses are true. A small integral 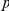-value for the hypothesis indicates that the 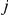-th regulator controlled at least one or more functional gene sets during the change of the modulator.

## Supporting Information

Figure S1 **Quantitative real-time RT-PCR analysis of KLF5 in siKLF5-treated A549 cells.**
(PDF)

Figure S2 **Expression profiles of miR-100 in order of ascending the EMT-related modulator values.**
(PDF)

Figure S3
**miR-100-induced changes in biologic characteristics in NCI-H1437 and NCI-H727 NSCLC cell lines.** (a) Representative phase contrast microscopic images showing negligible changes in morphology by miR-100 introduction in both NSCLC cells lines. NC#2, negative control. (b) Motility assay showing increased migration by introduction of miR-100 in both NSCLC cell lines. *, 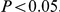

(PDF)

Figure S4
**Histogram of computational times for inferring cancer cell line-specific gene networks running on 12 core CPUs**. The 762 cancer cell line-specific gene networks related with the EMT were calculated from 13,508 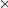 762 gene expression data when 100 target genes were randomly selected among 13,508 genes and the number of regulators was not restricted, i.e., 1,732 regulators were used. The comptational times were based on 12 core CPUs (Intel Xeon Processor E5450 (# of cores  = 4, clock speed  = 3.0 GHz)
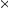
3). The histogram was calculated by 100,000 iterations.(PDF)

Figure S5
**Example of paths among four genes,** 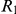 **,** 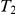 **,** 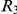 **, and** 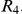

(PDF)

Table S1 **List of candidate regulators mapped to 1183 transcription factors and 47 nuclear receptors.**
(XLS)

Table S2 **List of candidate regulators mapped to 502 human microRNAs.**
(XLS)

Table S3
**List of coherent genes **(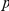 **-value** 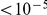 **) related to EMT calculated by extraction of expression module (EEM).**
(XLS)

Table S4 **EMT-related modulator values of 762 cancer cell lines calculated by signature-based hidden modulator extraction.**
(XLS)

Table S5 **List of 370 putative master regulators of E-cadherin during the EMT which were estimated by NetworkProfiler.**
(XLS)

Table S6 **List of 627 putative master regulators of E-cadherin which were estimated by a structual equation model (SEM) with the elastic net.**
(XLS)

Table S7
**Regulator function matrix between 1732 regulators and 5 functions.** The row and column indicate regulator and functional gene set, respectively. The (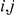)-th element represents the change during the EMT in the statistical significance (-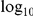
(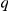-value)) for the enrichment of target genes of the 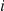-th regulator on the 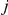-th function. The last column indicate the integral 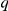-value of each row regulator which were used to determine which regulator strongly affected the functional gene sets.(XLS)

Table S8
**List of 17 putative master regulators (integral** 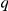 **-value** 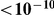 **) which correlated at least one or more EMT-related functions and were known to be downstream targets of TGFB1 with published evidence from Ingenuity Knowledge Base (** http://www.ingenuity.com **).**
(XLS)

Table S9 **List of the changes in the regulatory effects from 1732 regulators to E-cadherin and vimentin during the EMT.**
(XLS)
