# Supplementary figures and images for: A Novel Network Profiling Analysis Reveals System Changes in Epithelial-Mesenchymal Transition
Source: PLoS One. 2011 Jun 7;6(6):e20804. doi: 10.1371/journal.pone.0020804 (PMC3110206; doi:10.1371/journal.pone.0020804)

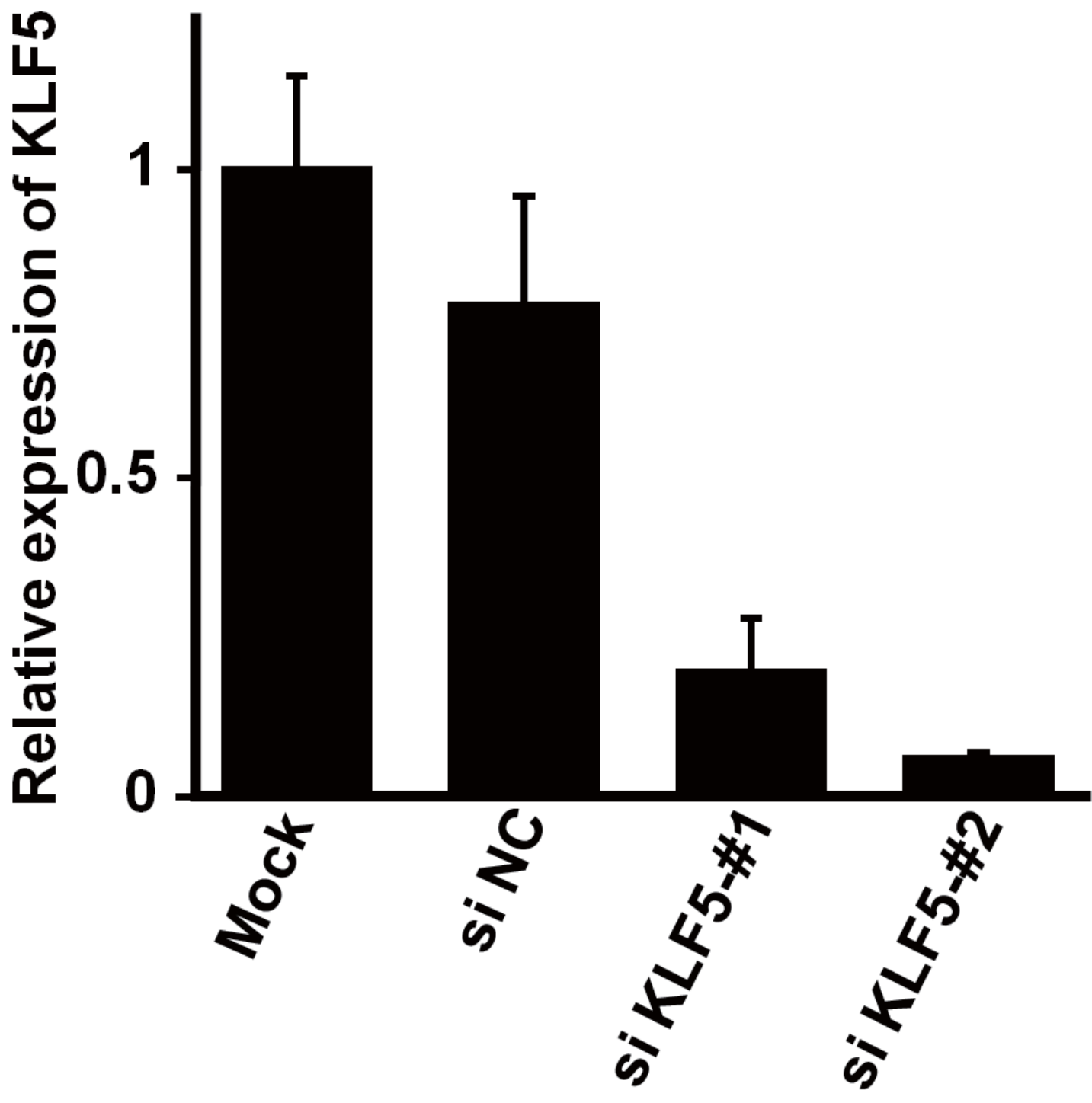

Supplement: Figure S1 — Quantitative real-time RT-PCR analysis of KLF5 in siKLF5-treated A549 cells. (PDF) [file pone.0020804.s001.pdf]

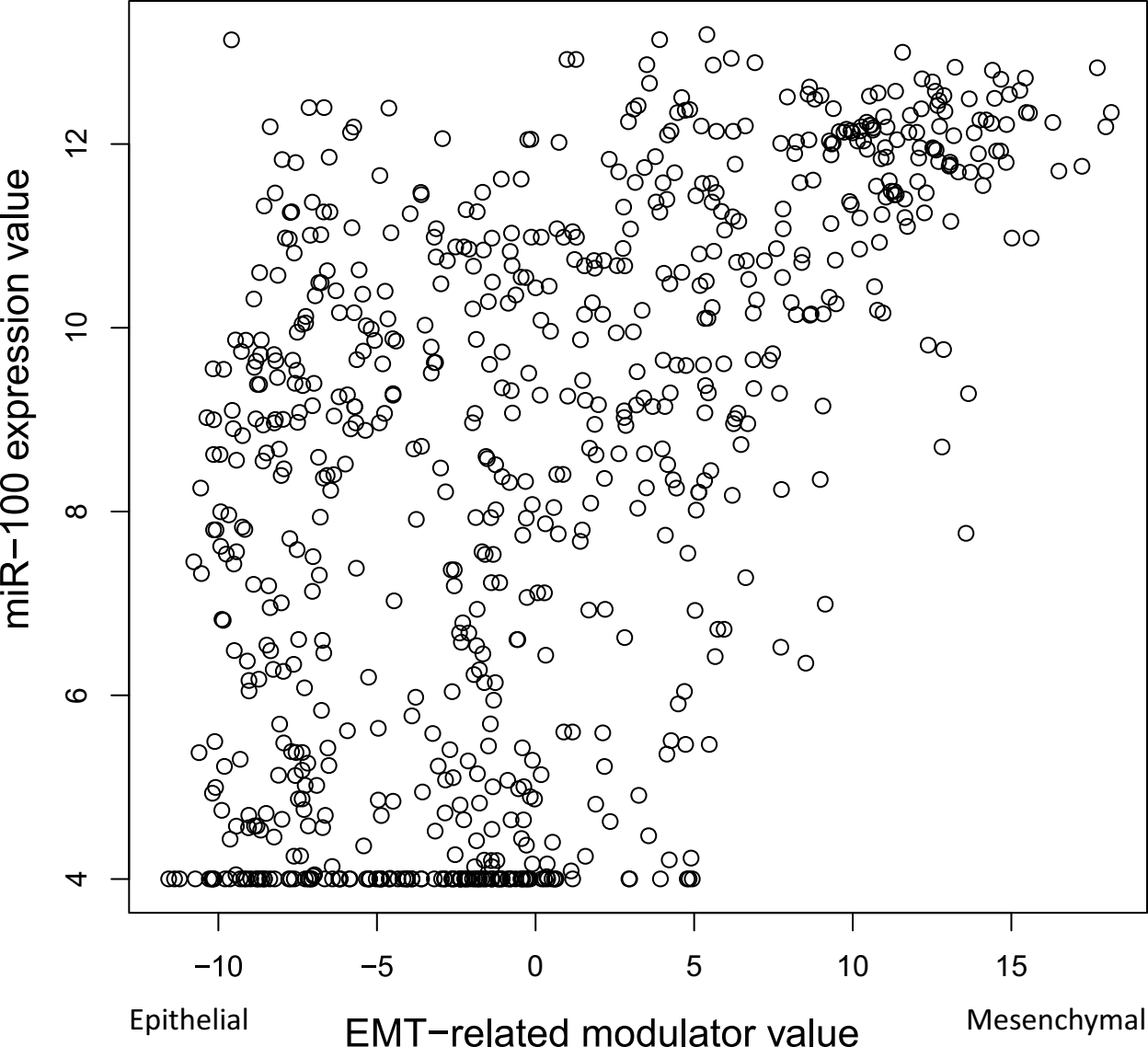

Supplement: Figure S2 — Expression profiles of miR-100 in order of ascending the EMT-related modulator values. (PDF) [file pone.0020804.s002.pdf]

**a****H1437****H727****NC #2**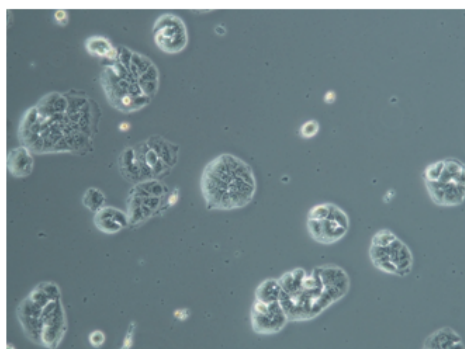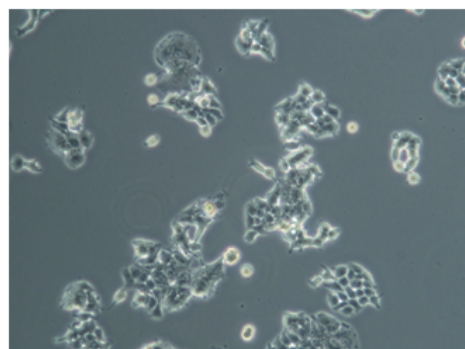**miR-100**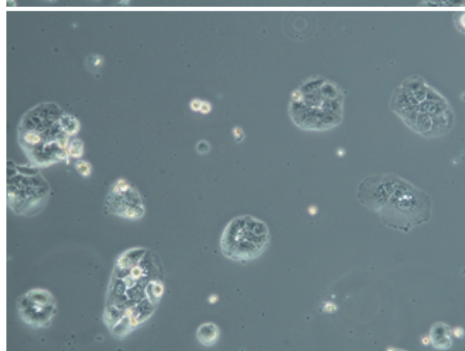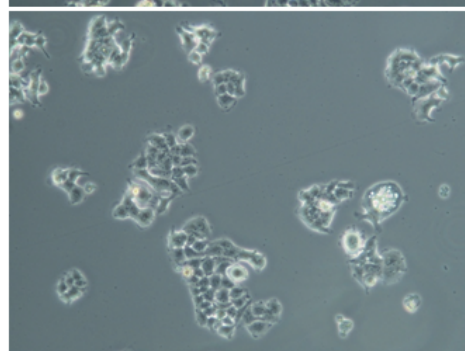**b**  
**Migrated cells (no.)****H1437****H727**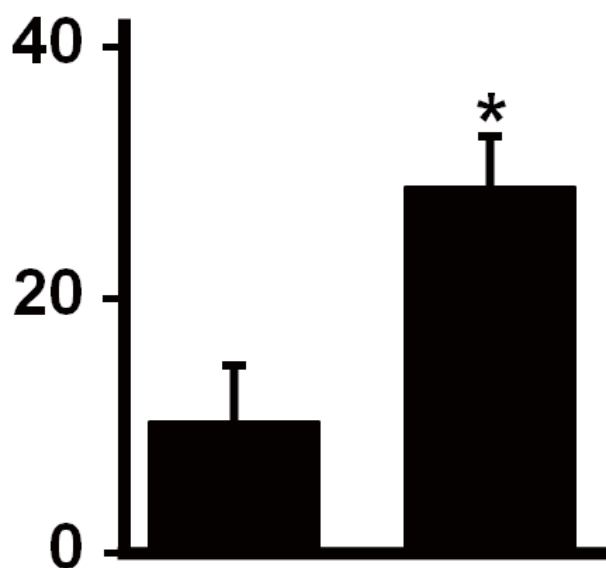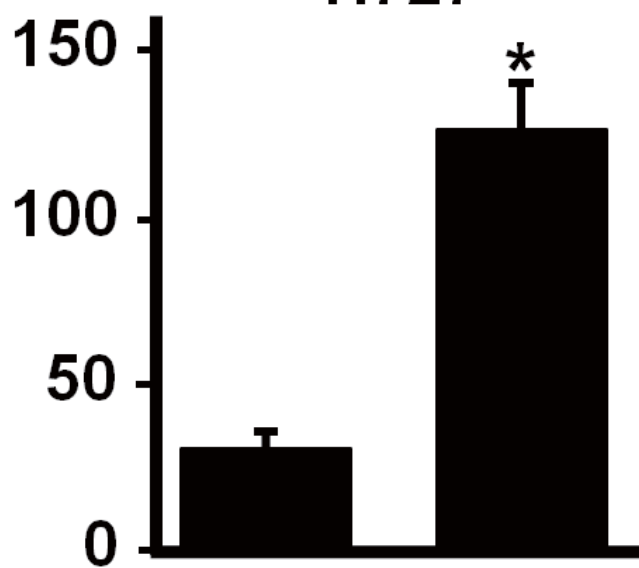**NC #2****miR-100****NC #2****miR-100**

Supplement: Figure S3 — miR-100-induced changes in biologic characteristics in NCI-H1437 and NCI-H727 NSCLC cell lines. (a) Representative phase contrast microscopic images showing negligible changes in morphology by miR-100 introduction in both NSCLC cells lines. NC#2, negative control. (b) Motility assay showing increased migration by introduction of miR-100 in both NSCLC cell lines. *, (PDF) [file pone.0020804.s003.pdf]

# Histogram of Calculation Time

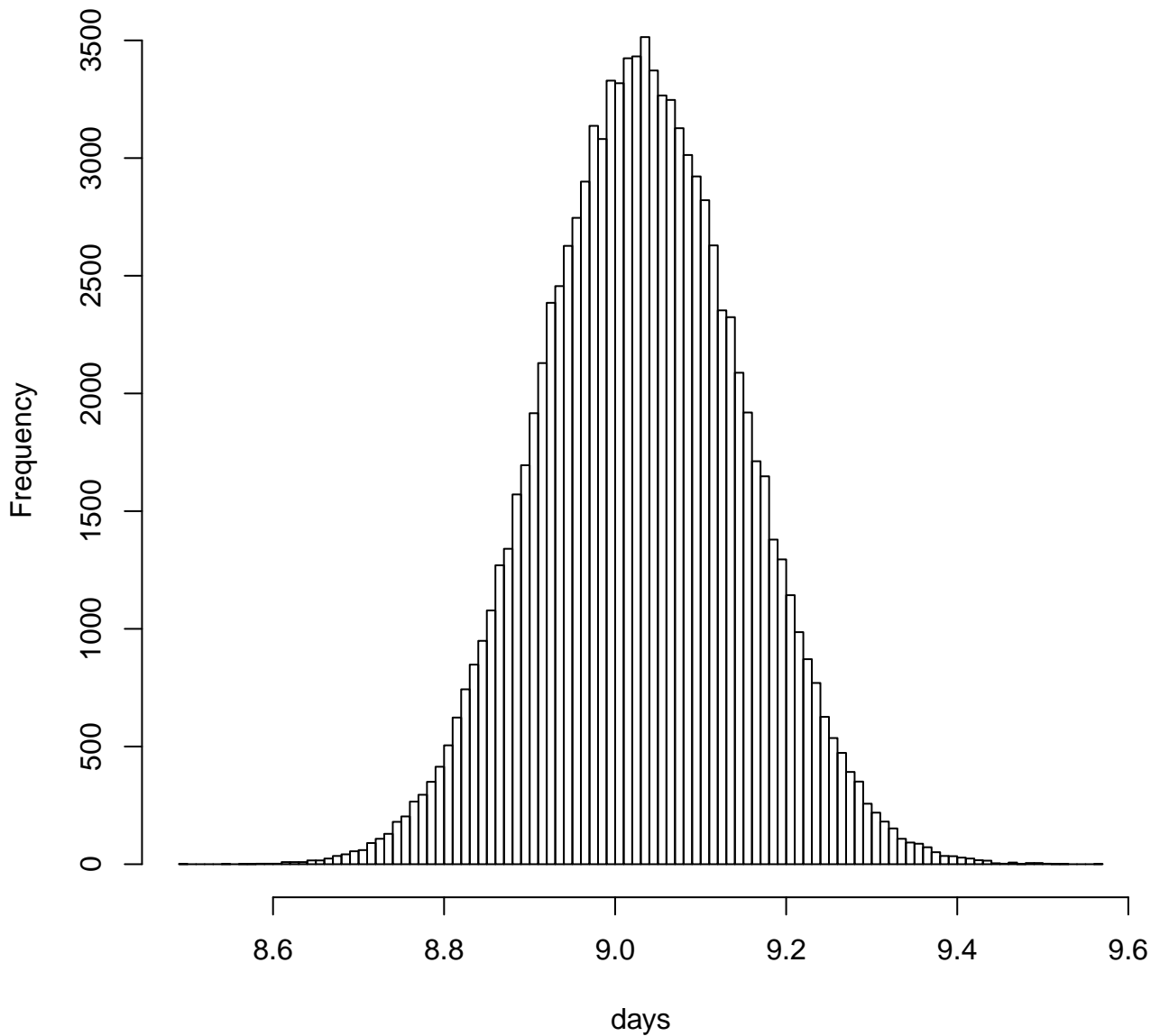

Supplement: Figure S4 — Histogram of computational times for inferring cancer cell line-specific gene networks running on 12 core CPUs. The 762 cancer cell line-specific gene networks related with the EMT were calculated from 13,508 762 gene expression data when 100 target genes were randomly selected among 13,508 genes and the number of regulators was not restricted, i.e., 1,732 regulators were used. The comptational times were based on 12 core CPUs (Intel Xeon Processor E5450 (# of cores = 4, clock speed = 3.0 GHz)3). The histogram was calculated by 100,000 iterations. (PDF) [file pone.0020804.s004.pdf]

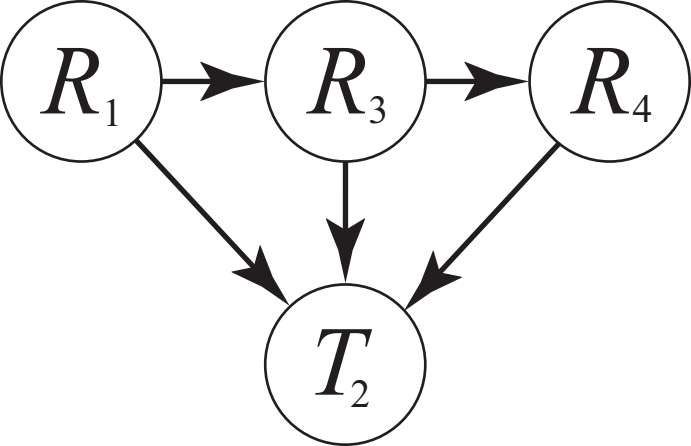

Supplement: Figure S5 — Example of paths among four genes, , , , and (PDF) [file pone.0020804.s005.pdf]
